# Principles of environmentally sustainable agriculture for building resilient and resource-efficient food systems

**DOI:** 10.55730/1300-0152.2764

**Published:** 2025-10-06

**Authors:** Ramazan ÇAKMAKÇI, Songül ÇAKMAKÇI, Muhammet Fatih ÇAKMAKÇI

**Affiliations:** 1 Department of Field Crops, Faculty of Agriculture, Çanakkale Onsekiz Mart University, Çanakkale, Turkiye; 2 Department of Food Engineering, Faculty of Agriculture, Atatürk University, Erzurum, Turkiye; 3 Department of Computer Programming, Vocational School, Mudanya University, Bursa, Turkiye; 4 Department of Computer Engineering, Graduate School of Natural and Applied Sciences, Atatürk University, Erzurum, Turkiye

**Keywords:** Sustainable food production, resilience, environmental sustainability, alternative farming techniques, synthetic biology, digital agrifood systems

## Abstract

As the demand for greater quantities of higher-quality food grows with population expansion, climate change, urbanization, and unsustainable agricultural practices accelerate the loss of arable land, ultimately threatening agricultural sustainability. Population growth necessitates a transition to nutritious, safe, and healthy food production systems that ensure higher yields, less reduced waste, improved social outcomes, and the integration of economic, social, and environmental sustainability principles. Urgent global challenges such as resource depletion, biodiversity loss, and climate change necessitate the protection of ecosystems and the sustainable use of natural resources. Agricultural systems must enhance food production and supply productivity, strengthen system resilience, and improve resource efficiency and sustainability. The sustainable development of agricultural systems based on resilience and productivity is important to ensure food security. The aim of this review is to compile, describe, and propose future strategies for promising food systems—including transformative innovations and alternative farming techniques—to facilitate the transition toward resilient, resource-efficient, and sustainable agriculture, and to mitigate long-term challenges. It also provides recommendations for future research, sustainability, resilience, and emerging food trends aimed at promoting sustainable food systems and green technologies, protecting ecosystems, resources, and biodiversity, and optimizing waste management and natural resource use. This article focuses on future sustainable food production and security, environmental protection, alternative protein sources, and innovative agricultural techniques; it highlights scientific and technological advancements, emerging research directions, and offers a comprehensive perspective on resilient, resource-efficient, and sustainable food production systems.

## 1. Introduction

Climate change and extreme weather events, population growth and rising food demand, environmental degradation, the misuse and depletion of natural resources, inequality, and, above all, sustainability and food security have become major global challenges, requiring more intensive and alternative production systems. As the global population continues to rise, food insecurity has become increasingly alarming due to biodiversity loss, ecosystem destruction, and threats to natural habitats; ensuring food security through sustainable farming techniques and practices has therefore become a pivotal strategy (Chowdhuri and Pal, 2025). Although the global food system has expanded rapidly over the last century—now covering more than 38% of the Earth’s habitable surface (FAO 2022a^1^)—approximately 2.4 billion people still experience moderate or severe food insecurity each year (FAO et al., 2023).

While this unsustainable expansion has temporarily improved food availability, it has simultaneously generated serious environmental challenges. Approximately 21%–37% of total global greenhouse gas (GHG) emissions originate from food systems (Shukla et al., 2019), with agriculture accounting for about 25% of GHG emissions (IPCC, 2020^2^), consuming roughly 70% of global freshwater, and contributing to over 80% of nitrogen and phosphate pollution in aquatic ecosystems (UNIDO, 2022^3^). The excessive use of nitrogen and phosphorus fertilizers and the widespread release of pesticides contribute to freshwater pollution and soil degradation, resulting in nearly one-third of the world’s agricultural land being classified as moderately to highly degraded (FAO, 2021).

Contemporary high-input, resource-intensive agricultural systems cause natural resource depletion, water scarcity, and severe environmental and social consequences. Modern industrial agriculture, characterized by high inputs and high outputs, has significantly increased production and food availability over time. However, these systems have also resulted in numerous adverse outcomes, including soil and environmental degradation, climate change, greenhouse gas emissions, nutrient pollution, global habitat and biodiversity loss, declining soil carbon stocks, habitat destruction, pest resistance evolution, reduced ecosystem and agricultural landscape resilience, decreased farm income and access to health services, and limited water storage and supply capacity—collectively posing severe risks to the environment and production systems (Hawkins et al., 2019; Chen et al., 2020; Jansen et al., 2020; Laborde et al., 2021; Ma et al., 2021; Prăvălie et al., 2021; Bras et al., 2022; Dirzo et al., 2022; Çakmakçı et al., 2023).

Despite higher productivity, the current industrial agrifood system remains unsustainable and inequitable because of its environmental impacts and its failure to provide healthy nutrition for people. Industrial agriculture has compromised air, water, and soil quality while driving habitat destruction, natural resource depletion, agricultural system deterioration, and unsustainable growth trends in food production (Fatima et al., 2024). This system contributes to the climate crisis through excessive water and land use, pesticide pollution, biodiversity loss, and land degradation (Díaz et al., 2019; Qu et al., 2024). Current agricultural, food production, and consumption systems are unsustainable for both human and planetary health (Alae-Carew et al., 2022), leading to continued resource depletion and severe environmental and social consequences (Hebinck et al., 2021). Moreover, the spatial separation of crop and livestock production in high-input commercial agriculture creates significant sustainability challenges, including climate change, nutrient imbalances, biodiversity loss, water pollution, and the growing vulnerability of rural livelihoods (Garrett et al., 2020). The dependence of current food systems on a limited number of edible plant species and varieties also threatens both food and nutritional security (Zuza et al., 2024).

Since the green revolution, agricultural expansion, mechanization, and the intensive use of chemical fertilizers, pesticides, and genetically improved high-yielding crop varieties have become widespread, leading to large-scale overproduction that is increasingly costly and environmentally damaging (Qu et al., 2024). Overreliance on energy-intensive industries has raised major environmental concerns (Luo et al., 2024). Efforts to ensure food security, together with the ever-increasing use of agrochemicals and production growth initiated by the green revolution, have also resulted in negative economic, environmental, and social impacts. Massive overproduction has become progressively more costly and environmentally detrimental (Giller et al., 2021). Environmental problems, climate change, diminishing resources, and the low productivity and profitability of farms adversely affect the food security and sustainability of agricultural production systems (Fatima et al., 2023). Enhancing agricultural productivity is essential to meet rising food demands, improve global living standards, and ensure the sustainability of food production. However, increasing crop productivity also entails risks such as global warming, reduced arable land and microbiome biodiversity, water scarcity, excessive fertilizer and pesticide use, and environmental pollution. Agrifood systems have a substantial environmental footprint and both influence and are affected by climate change (Campbell, 2022), biodiversity loss (Daskalova et al., 2020), land-use change, soil degradation, and water pollution (OECD, 2019).

Although increasing crop productivity and achieving higher yields per unit area are considered solutions to ensuring an adequate food supply, such gains typically require greater resource consumption, including agricultural chemicals, mineral fertilizers, and nonrenewable resources such as fossil fuels. The excessive use of resources in high-efficiency agriculture has resulted in numerous environmental imbalances. These include pollution, soil loss, alterations in wildlife populations, and shifts in local flora and fauna, all of which negatively affect ecosystems and threaten sustainability (Gamage et al., 2024). Growing concerns about the adverse impacts of intensive farming practices have spurred the demand for more sustainable agricultural systems. Efforts to ensure food security and environmental sustainability have ushered in a transformative era of agriculture that integrates new technologies while addressing ecological concerns (Gamage et al., 2024). Because current production and development models that overexploit resources and generate excessive waste also cause socioeconomic imbalances, a transformation toward sustainable, nature-based agrifood systems that embrace agroecology for food sufficiency and security has become essential. Although current food systems may be able to feed the global population in the coming decades, substantial changes are clearly required to enhance sustainability, reduce food waste, and establish nutritional and production patterns that are both environmentally sustainable and beneficial to human health.

According to the FAO’s definition, food security is “a situation in which all people, at all times, have physical and economic access to sufficient, safe, and nutritious food to meet their dietary needs and preferences for an active and healthy life” (FAO et al., 2023). Although the fundamental components of food security are availability, access, utilization, and stability (Çakmakçı et al., 2024), the first pillar—food availability—is primarily dependent on agricultural production (Bahn et al., 2021). There is an urgent need for holistic and integrated approaches in food production systems aligned with the sustainable development goals (SDGs), along with the effective utilization of available technologies and resources.

## 2. Sustainable agriculture and food systems

According to the FAO (2021), a sustainable food system is one that “ensures food security and nutrition for all in such a way that the economic, social, and environmental foundations for generating food security and nutrition for future generations are not compromised”. Food safety management encompasses food, agriculture, economics, and environmental sciences (Shen et al., 2021). Food security, which encompasses the availability, accessibility, and purchasing power of individuals to obtain sufficient, safe, culturally acceptable, and nutritionally adequate food to meet dietary needs for a healthy life (Oyelami et al., 2023), emphasizes quality as much as quantity (Çakmakçı and Çakmakçı, 2023a).

**Figure 1:**
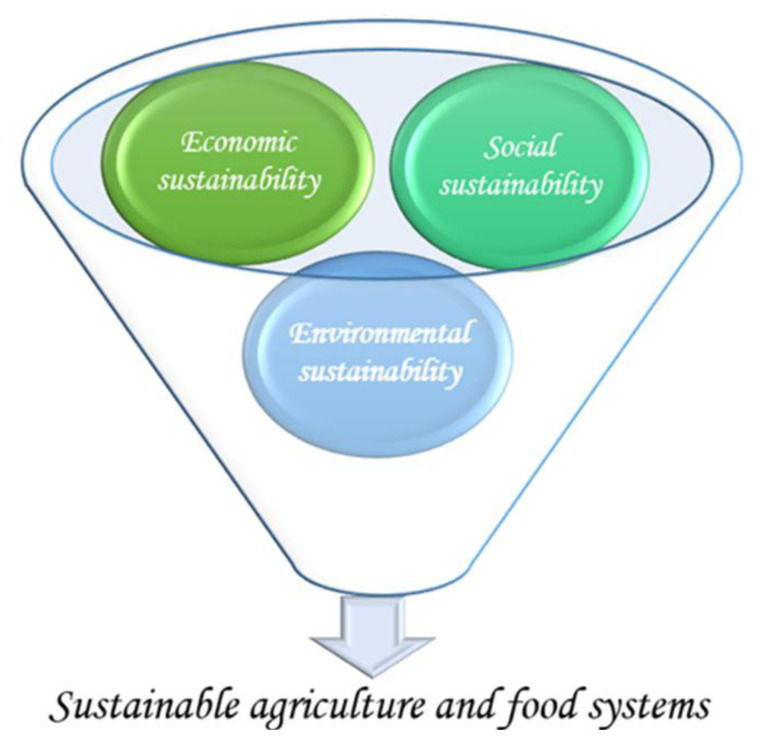
The three dimensions of sustainability in agriculture and food systems.

Sustainability should ensure and promote the integration of all three pillars—environmental, social, and economic—of sustainable development (Figure 1). A socially, economically, and ecologically sustainable food system is generally defined as one capable of mitigating the impacts of climate change, maintaining ecosystem health, reversing biodiversity loss, and producing sufficient, safe, and nutritious food for present and future generations (Çakmakçı et al., 2023). Given the challenges posed by limited resources, innovation and technology must be developed to promote sustainability in the agricultural sector, grounded in environmental health, financial viability, and social equity (Gamage et al., 2024).

The primary condition of sustainability—which must also encompass economic and social dimensions—is ecological sustainability; long-term economic development should occur within the limits of natural resource availability and renewal capacity (Pham et al., 2020). Economic sustainability refers to the capacity of a system to adapt to changing conditions, sustain itself, and manage resources effectively to support current and future generations (Liang et al., 2024). Sustainable agriculture is founded on site-specific practices that primarily target environmental health, financial viability, and social equity, aiming to meet the needs of both present and future societies. The imperative to achieve regional food self-sufficiency has become particularly critical (Lu et al., 2024).

More localized agricultural production offers advantages in reducing transport costs and associated emissions, while supporting diverse farming systems and rural livelihoods (Giller et al., 2021). Local food production is a recommended approach to promoting food system sustainability and revitalizing communities, enhancing environmental security, ecological integrity, social equity, and the capacity of smallholders to compete with large-scale food distribution networks (Kujala and Koppelmäki, 2024). Beyond mitigating the impacts of increased competition and extended food chains, the promotion of value-added local products, regional resource utilization, local consumption, and the valorization of agricultural byproducts, food wastes, and local biodiversity contribute to building a sustainable and environmentally friendly agrifood system (de Vries et al., 2022). Local and regional agrifood systems create spaces for direct contact and interaction between producers and consumers. Indigenous species play a vital role in providing high levels of essential nutritional compounds. Local agrifood systems (LAFSs) contribute to enhanced food security, reduced environmental impacts, and improved nutrition (Burnett, 2023).

Sustainable agriculture fundamentally integrates natural biological cycles and control mechanisms; maintains and restores soil fertility and the natural resource base; minimizes dependence on nonrenewable resources and external inputs; optimizes input efficiency; ensures adequate and stable agricultural income; and mitigates adverse impacts on human health, wildlife, food safety, water quality, and the environment. This system aims to balance human nutritional needs with environmental protection, resource conservation, economic resilience, social equity, and the long-term sustainability of agricultural production (Dönmez et al., 2024). Sustainability seeks to balance the economic, environmental, and social dimensions of agricultural production, fostering a resilient agricultural system while promoting the stability and continuity of productive farming landscapes (Rose et al., 2019). The environmental dimension of sustainability involves protecting and enhancing environmental quality by reducing the use of nonrenewable resources, preventing air, soil, and water pollution, and promoting biodiversity (Shahmohamadloo et al., 2022). Renewable energy sources such as wind, solar, and hydroelectric power contribute significantly to environmental sustainability (Luo et al., 2024).

While the environmental goals of sustainable food systems include reducing water, carbon, nitrogen, phosphorus, energy, land, ecological, and biodiversity footprints, their socioeconomic objectives focus on increasing agricultural output, improving economic performance, achieving social equity, reducing food costs, and incorporating dietary preferences (Niu et al., 2024). Sustainable agriculture, as a holistic strategy, encompasses practices and technologies that conserve resources, remain economically viable and socially inclusive, and mitigate environmental harm while ensuring farm profitability and food security. The transition to a sustainable food system requires the generation and adoption of new knowledge and technologies, collectively referred to as sustainability-oriented innovation, green innovation, and eco-innovation (Little et al., 2023). Sustainability-oriented innovation is defined as the development and dissemination of new or improved social and technological knowledge that transforms production and consumption systems, enhances community well-being, and creates social and environmental value while generating economic returns (Testa et al., 2022; Little et al., 2023).

Crop diversification and the integration of mixed crop–livestock systems contribute to economic sustainability by enhancing farm resilience and profitability. The concept of sustainable development has gained increasing importance in research and policymaking related to the management of environmental issues and industrial and agricultural production, aiming to safeguard the planet’s living systems (Ruggerio, 2021). Sustainable development is defined as development that meets the needs of the present without compromising the ability of future generations to meet their own needs. A key driver of progress toward achieving sustainable development goals in agriculture is expected to be regionally focused policies that influence input use, agricultural production, and the mitigation of agriculture’s negative environmental impacts (Kosari-Moghaddam et al., 2025).

## 3. Resilience and sustainability in agricultural systems

Resilience refers to the ability to manage and cope with change—the capacity of systems to respond and reorganize in ways that preserve their core functions and identities in the face of adversity (Knickel et al., 2018). It describes the capacity of a system to adapt, recover, and continue providing its functions during and after periods of disruption (Núñez-López et al., 2022). Increasing land and water scarcity, inequality, competition over resource use, soil degradation, and biodiversity loss are exacerbated by climate change, resulting in reduced diversity and productivity, thereby making agricultural systems more vulnerable (Meybeck et al., 2024). Water scarcity intensifies land scarcity, while the overexploitation and consumption of groundwater resources beyond their natural renewal and recharge rates lead to severe water shortages. Indeed, a sustainable and resilient agricultural system is expected to enhance production and resource-use efficiency, reduce environmental impacts, and contribute positively to climate stability, biodiversity conservation, soil quality, and persistence under extreme weather events. Agricultural systems play a vital role in building resilience through practices that promote conservation, ecological restoration, and biodiversity (Meybeck et al., 2024).

Because of their complexity, food systems are highly vulnerable to a wide range of shocks and stresses (Fan et al., 2021). Extreme weather events, disease outbreaks, changes in land and agrochemical use, dietary shifts, pollution, demographic and regulatory changes, fluctuations in commodity prices, reduced pollinator and beneficial microorganism activity, and biodiversity loss can all significantly affect food systems (Zurek et al., 2022; Çakmakçı, 2024). A resilient agricultural production system capable of withstanding shocks can be achieved through the adoption of innovative technologies (Usigbe et al., 2024). Crop production integrating various AI-based technologies ensures optimal yields at minimal cost, thereby contributing to agricultural sustainability and strengthening resilience within food systems (Shaikh et al., 2022; Uyeh et al., 2023). Diversity in protein sources—such as legumes, insects, algae, and microbes—along with diversity in land use and plant species, are key determinants of food system resilience (Kahiluoto, 2020).

As a dynamic and cross-sectoral concept, resilient food systems are defined as “the capacity of a system and its components to anticipate, absorb, and recover from the impacts of disturbances while maintaining, restoring, or improving their essential structures or functions” (Sperling et al., 2022). Ecological resilience refers to the capacity of an ecosystem to withstand disturbances without altering its self-organizing processes and structural integrity. Resilient agricultural systems must be capable of absorbing disruptions and reorganizing themselves in response to changing conditions. Ensuring resilience in rural development requires the protection and sustainable management of all natural resources, the optimization of their use, and the enhancement of resource efficiency. Sustainable agricultural systems should promote a certain degree of self-regulation, reflecting nature’s inherent levels of organization, resilience, and resistance. First, when nonnative species are introduced into an ecosystem, native species should not be adversely affected.

Integrated nutrient and water management, adjusting planting dates based on soil moisture conditions (Kamdi et al., 2023), and cultivating alternative crops (Islam et al., 2023) have been proposed to enhance food security and health, strengthen resilience to climate variability, and restore ecosystems. Given the sustainability challenges facing agricultural systems under climate change, transforming agriculture into an ecologically sustainable, climate-resilient agriculture (CRA) management model has become increasingly important (Zong et al., 2022). Alternative systems such as climate-smart regenerative agriculture—which integrates biotechnology, engineering, and ecological farming technologies in harmony with natural processes—are needed to enhance resilience to climate change, improve sustainable productivity, and strengthen ecological, economic, and social dimensions of food security (Keshavarz and Sharafi, 2023).

Climate-resilient food systems require conservation agriculture (Sgroi, 2022), agroforestry (Barrios et al., 2020), and integrated soil and water conservation practices (Duc et al., 2021). Compared to conventional agricultural systems, agroforestry systems demonstrate greater resilience to environmental shocks and climate change. Diversified and integrated farming systems that enhance resource efficiency and resilience, along with sustainable biobased economy and landscape approaches, have been proposed to achieve more inclusive and resilient agrifood systems (Meybeck et al., 2024). Building resilience in food systems and ensuring food security require strategies of resilience, recovery, and reorientation (Zurek et al., 2022). Diversification of food systems, including the use of diverse tree species, is urgently needed to enhance resilience at both local and global scales (Jansen et al., 2020). Introducing neglected and underutilized species—whether wild, cultivated, or semidomesticated—into agricultural systems can enhance the resilience and sustainability of food production (Ulian et al., 2020). The development and introduction of neglected and underutilized crops adapted to regional and local conditions—and promoting climate change adaptation—are crucial for building resilience and strengthening food systems (Chimonyo et al., 2022).

Resilience requires the use of resistant crop varieties, effective water storage, the provision of adequate natural habitats and field boundary features to support pollinators, crop rotation, diversified supply chains, and the maintenance of soil quality and nutrient reserves (Hess et al., 2020; Zurek et al., 2022). Conversely, disruptions in food supply and food security necessitate strengthening the resilience of food production systems and reducing dependence on imported inputs (Rikkonen et al., 2024). Restoring degraded areas, protecting resources, and ensuring sustainable marine protein supplies are essential for restoring biodiversity and building more resilient and sustainable food systems (Sperling et al., 2022).

Agricultural biodiversity—including the diversity of crop species and varieties, livestock breeds, and beneficial native plants and pollinators—enhances the resilience, productivity, and adaptability of food systems to climatic and economic shocks while reducing associated risks (Zuza et al., 2024). The sustainable intensification of cropping systems enhances resilience to climate change impacts, strengthens food security, and promotes environmental sustainability (Yadav et al., 2024). Reliance on a limited number of species in food production and the expansion of monocultures are displacing local foods and traditional practices, thereby increasing food insecurity and systemic risks. Forests that enhance agricultural resilience are increasingly being degraded and destroyed. Biodiversity loss results in soil and water degradation, diminished resilience to climate change, heightened vulnerability to pests and diseases, and growing food and nutrient insecurity. Diverse crop types and cropping systems protect local economies by ensuring stable markets, align with ecological practices, and form the foundation of community-supported agriculture (Zuza et al., 2024). Species-rich ecosystems have been shown to exhibit greater resilience, reduced dependence on chemical inputs (Wang et al., 2023), stronger resistance to climatic stressors (Maxted and Brehm, 2023), and enhanced overall environmental stability (Eisenhauer et al., 2023). Crop species diversity contributes substantially to soil health and water management by optimizing nutrient cycling, enhancing soil structure, increasing organic matter content, and promoting microbial diversity. Perennial ground cover systems—considered a cornerstone of agricultural sustainability—can enhance water and nutrient use efficiency, increase microbial biomass and diversity, and strengthen agricultural resilience and food security (Schlautman et al., 2021).

For urban populations, ensuring access to sufficient and nutritious food in the face of shocks is a fundamental component of resilience. Local production and short supply chains can reduce the likelihood of food supply disruptions and dependence on external inputs, while source diversification can provide “spare” capacity, thereby improving the ability of food systems to respond and adapt to shocks (Gulyas and Edmondson, 2021). The most evident impact of urban and peri-urban agriculture (UPA) on city resilience is its contribution to household and urban food security by supplying locally produced fresh foods, particularly to meet micronutrient requirements (Gulyas and Edmondson, 2021). UPA contributes to the social, economic, and environmental sustainability of urban food systems, ultimately enhancing urban socioecological resilience (Kong et al., 2025).

## 4. Climate-resilient microbial biotechnology for sustainable agriculture

The main challenges to environmental sustainability include the production and use of synthetic pesticides and chemical fertilizers, as well as the release of methane and other pollutants from farms, which lead to the accumulation of harmful compounds in soil and water bodies. Green microbiology offers pathways to “environmental sustainability” through clean production, effective waste management, renewable biofuels, nutritious and sustainable foods, the biodegradation of harmful wastes into less harmful compounds, and the production of clean and renewable energy (Akinsemolu, 2023). The ability of microorganisms to survive in extreme conditions, adapt to environmental changes, and reproduce rapidly (Kochhar et al., 2022; Thakur et al., 2022) is crucial for food production and environmental protection, as these abilities can be harnessed to promote environmental sustainability. Promising microbe-based solutions are being developed, including the biodegradation of plastics (Heris, 2024), the production of bioethanol from xylose and glucose (Talapko et al., 2022), and the use of algae as protein sources (Diaz et al., 2023) for biomass generation through photosynthesis and wastewater purification (Kube et al., 2022).

Microbial communities maintain the structure, function, and dynamic stability of ecosystems; they also enhance plant interactions, strengthen agricultural immunity, and significantly influence sustainability (Tan et al., 2022). To reduce soil pollution in agricultural ecosystems and maintain ecological safety, strategies such as developing environmentally friendly microbial fertilizers, biocontrol bacteria, and agricultural microbial agents have been proposed, based on insights from plant-microbe interactions and advances in agricultural microbiology (Basu and Kumar, 2020). Beneficial microorganisms can be isolated, genetically engineered to express desired traits, and subsequently inoculated into the plants. Crops can be cultivated without chemical inputs by harnessing the plant microbiome, which enhances plant sustainability and disease resistance. Microbe-based bioproducts provide a cost-effective, pollution-free, and sustainable approach to mitigating biotic and abiotic stresses induced by global climate change, enhancing agricultural biodiversity, and supporting clean food production. Alternatively, transitioning to sustainable food systems—such as those incorporating plant-based meat and dairy alternatives—is recommended to meet global climate change mitigation targets (Alae-Carew et al., 2022). Species diversity, which enhances ecological resilience, remains a critical determinant in transforming food systems (Zuza et al., 2024). Species diversity within agricultural systems improves water quality and ecosystem health and can significantly reduce the environmental footprint of food production.

The bioeconomy, which relies on the recycling and reuse of resources, is grounded in the fundamental principles of sustainable consumption and resource management, resilient and diverse ecosystems, and food security, forming a core component of environmental sustainability (Akinsemolu, 2023). Microorganisms not only provide inexpensive and effective nutritional supplements but also, through their participation in biogeochemical cycles, play a crucial role in maintaining ecosystem resilience and diversity—core principles of the bioeconomy. Green microbiology offers key benefits, including driving the production of sustainable alternatives to products that deplete the renewable capacity of natural resources and developing microbe-based products for essential needs such as food and energy (Akinsemolu, 2023). As resource scarcity and environmental degradation intensify, energy conversion technologies, the utilization of biomass as a renewable energy source, and innovative waste management strategies are gaining importance as viable and promising solutions.

## 5. Assessment of agricultural sustainability

Sustainable food systems that ensure food security and nutrition for all—both now and for future generations—enhance productivity, promote the consumption of local products and short supply chains, and protect the environment. A sustainable agricultural system emphasizes the efficient use of renewable resources and inputs; minimizes environmental impacts; promotes biodiversity; improves soil structure and health; enhances adaptive capacity to climate change and variability; employs integrated pest management and crop diversification; supports local communities and labor; targets long-term sustainability; integrates traditional knowledge with innovative technologies and practices; and ensures food quality and safety (Çakmakçı et al., 2023, Çakmakçı et al., 2024; Dönmez et al., 2024). Innovative agricultural technologies, adaptive capacity, environmental and economic sustainability, and social responsibility constitute the key components of inclusive, equitable, and resilient sustainable food production systems (Qu et al., 2024). Sustainable agriculture aims not only to ensure environmental health, resource efficiency, and socioeconomic sustainability but also to secure a livable future for agriculture while maintaining current productivity levels.

The assessment of agricultural sustainability focuses on integrating ecological, socioeconomic, and environmental dimensions and optimizing the use of agricultural resources, particularly land and water (Sarkar et al., 2021). Sustainable farming encompasses agronomy, ecology, economics, and social sciences, promoting systemic solutions that increase crop yields while improving soil health and biodiversity by integrating innovations such as precision agriculture (PA) with ecological principles (Chowdhuri and Pal, 2025). There are four key pillars of concern for sustainable food systems, namely climate stability, biodiversity conservation, natural resource preservation, and the protection of clean air and water (Hansson et al., 2024). Actionable principles for research and innovation in sustainable agrifood systems (SAFS) have been proposed, including establishing a clear theory of change; designing and implementing transparent, evidence-based innovation processes; addressing synergies, efficiencies, and unintended consequences; ensuring food and nutritional security; managing natural resources responsibly; contributing to the economy; and developing an ethical, equitable, and adaptable agrifood system (Zurek et al., 2023).

## 6. Transitional pathways toward sustainable, resilient, and resource-efficient food systems

The growing fragility of agrifood systems has intensified the need for a transition toward sustainable, resilient, fair, and equitable agrifood systems. Persistent challenges—including poverty, inequality, the interlinkage of natural resources and biodiversity with livelihoods, and climate change—are recognized as major obstacles to achieving resilient and inclusive transformation in agriculture. A sustainable, resilient, just, and equitable food system must possess the capacity to ensure the continuous provision of sufficient, appropriate, and accessible food for society, despite various foreseeable or unexpected events and disruptions over time (Haller et al., 2022; Knickel et al., 2018). A resilient food system should ensure food and nutrition security under all possible circumstances (Kahiluoto, 2020).

Building resilient food systems and promoting rural transformation require practical strategies such as integrating traditional on-farm food systems into modern agriculture, diversifying household enterprises, strengthening youth employment capacity, providing business mentoring, and offering financial and technical assistance for startups (Schneider et al., 2024). Technological change can enhance living standards and overall welfare through increased productivity; however, it should not be overlooked that technologies may also exacerbate or reduce existing inequalities. Indeed, farmers with the resources and knowledge to implement agricultural technologies—and who adopt them early—often benefit disproportionately, thereby exacerbating inequalities (Arslan et al., 2022). Household enterprises play a critical role in transforming agrifood systems; however, they must be strengthened by enhancing the productivity and profitability of micro-farms (Rud and Trapeznikova, 2021).

Soil, water, and plant management—along with crop production—constitute key interconnected components and strategies of sustainable agriculture aimed at meeting current and future food demands while maintaining ecosystem viability and health. As key elements of sustainability, the sustainable management of water and land resources, as well as carbon, nitrogen, and phosphorus inputs, must be prioritized (Niu et al., 2024). Due to both excessive and inadequate fertilizer and resource management practices that deteriorate soil health and reduce crop yields, sustainability challenges in agriculture are becoming increasingly urgent. The sustainability of production systems requires optimizing planting structures, adjusting crop distribution, diversifying production modes, improving the utilization efficiency of agricultural and by-products, nitrogen fertilizers, and water resources, and adopting green energy solutions (Niu et al., 2024).

The bioeconomy holds significant potential for developing resilient and resource-efficient food production systems through the efficient use of resources, the economic valorization of biomass, and increased income generation (Schneider et al., 2024). To make food systems more sustainable and resilient, resource-efficient strategies such as industrial symbiosis (IS) are required, wherein food waste and by-products are reused as raw materials for other processes, thereby reducing the need for land, agrochemicals, transportation, and energy (Haller et al., 2022). Industrial symbiosis can contribute to sustainable food production and enhance system resilience by promoting more economical, circular, and equitable resource use.

Reducing the negative effects of intensive agricultural practices depends on improving input-use efficiency through methods such as PA and redesigning systems to substitute external inputs and promote ecosystem services (Soulé et al., 2023). An urgent and innovative transformation toward sustainable food systems is needed to ensure the food security of agrifood networks and minimize their negative environmental impacts (Herrero et al., 2020). Promising solutions, potential pathways, and innovations for improving agricultural efficiency and facilitating the transition to sustainable agriculture should be identified and developed to ensure food security (Table 1). These solutions include agroecological approaches, sustainable intensification, precision agriculture, integrated pest management, conservation agriculture, and sustainable soil and water management practices (Chowdhuri and Pal, 2025). Integrated crop–livestock systems—essential for human well-being—and diversified agricultural production systems across time and space should be adopted as sustainable models of agriculture (Garrett et al., 2020).

Sustainable crop production requires strategic planning, effective management, and the implementation of appropriate measures to enhance agricultural efficiency and ensure the sustainable use of agricultural lands. Ensuring the long-term sustainability of agricultural ecosystems requires the provision of technical support and knowledge, the encouragement of innovation, and the strengthening of training and partnerships between farmers and scientists (Gamage et al., 2024). Sustainability in agriculture and food systems requires enhancing productivity and climate resilience, reducing agricultural greenhouse gas emissions and land-use changes, and improving nutrition (Movilla-Pateiro et al., 2021). Sustainable development necessitates equitable access to resources, emerging markets, and innovative technologies; reducing food loss and waste; promoting healthy dietary transitions; and ensuring effective governance and support mechanisms.

Perennial groundcovers—also referred to as living mulches or perennial cover crops—protect soil health and natural resources while enhancing the resilience and food security of agricultural ecosystems (Schlautman et al., 2021). Various water-efficient techniques—including agroforestry (Wijerathna-Yapa and Pathirana, 2022), rainwater harvesting (Zhang et al., 2021), drip and subsurface irrigation (Liu et al., 2023; Demirel et al., 2020), cover cropping and mulching, and soil moisture monitoring (Çakmakçı and Çakmakçı, 2022)—play critical roles in sustainable agricultural practices. Perennial groundcover systems enhance water-use efficiency, stimulate microbial activity, and increase productivity and profitability by reducing dependence on external inputs (Schlautman et al., 2021). Alternating wetting and drying irrigation regimes—designed to reduce water input and use by 34%–70% and to improve water-use efficiency while maintaining grain yields—have successfully enabled rice cultivation without yield loss (Mote et al., 2023). Furthermore, specific strategies have been developed to enhance soil quality and promote sustainable potato cultivation (Sheng et al., 2023).

## 7. Approaches for building resilient and sustainable agricultural production systems

Numerous approaches, farming practices, and agricultural systems for sustainable production, environmental protection, and food safety have been proposed by researchers (Table 2). These include agroecology, agroforestry systems, organic and biodynamic farming, nature-inclusive and nature-positive agriculture, locally adapted PA, digital future farming, conservation and carbon agriculture, permaculture, regenerative and agroecological farming, sustainable land management, climate-smart agriculture, diversified farming systems, ecological intensification, integrated crop–livestock systems, forestry systems, integrated nutrient and water management, integrated pest management, double and multiple cropping, intercropping, and mixed and relay cropping (Mustafa et al., 2019; Muhie, 2022; Sperling et al., 2022; Boix-Fayos and de Vente, 2023; Çakmakçı et al., 2023; Guareschi et al., 2023; Osei et al., 2023). Adaptation strategies related to agricultural sustainability—such as agroforestry, crop diversification, the use of biofertilizers, intercropping, water-use efficiency, and agricultural conservation—help mitigate risks in crop production (Chowdhuri and Pal, 2025). Diversified agriculture, which integrates multiple species within a farm system, promotes ecological interactions, utilizes both planned and associated biodiversity, and relies on ecological processes that sustain production—such as ecological intensification, nutrient cycling, and biological pest management (Garibaldi et al., 2017).

Nature-based solutions (NBSs)—inspired by, supported by, or modeled after nature—are gaining increasing attention as actions that address societal challenges through the use of ecological processes to adapt to climate change and achieve the sustainable development goals (SDGs) (Albert et al., 2021). Nature-based solutions that harness ecosystem processes have the potential to protect ecosystems, resources, and biodiversity; mitigate and adapt to climate change; and reduce disaster risk. The natural assets that underpin agriculture are indispensable for life and form the foundation of agricultural systems that preserve biodiversity (Demirel et al., 2025).

Amid a growing population, water scarcity, and increasing food demand, alternative agricultural methods and techniques that are productive, resource-efficient, and resilient to changing climatic and weather conditions are urgently needed (Taheri et al., 2025). Alternative farming techniques based on agroecological principles—from soil health and water management to the use of natural inputs and biodiversity—aim to protect and enhance the natural resource base and the environment; increase productivity; provide farmers with profitability and energy savings; improve food quality, safety, and security; achieve long-term sustainability; and foster a vibrant socioeconomic infrastructure. Sustainable farming practices aim to enhance environmental quality and use resources efficiently to produce agricultural products that ensure food availability for future generations at minimal environmental cost (Sharma et al., 2024). In contrast to the depletion of soil functions caused by modern agricultural intensification, farm sustainability relies on enhancing soil fertility, structure, and biodiversity; increasing soil organic matter, and improving overall soil health. Sustainable agriculture depends on renewable resources; the conservation of natural capital; production at low environmental cost and high resource efficiency; site-specific management; the integration of crops and livestock; improvement of the environmental and natural resource base; and the adoption of environmentally friendly agricultural technologies (Muhie, 2022; Chen et al., 2024). To achieve sustainability by conserving resources and promoting ecological resilience in agriculture, integrated pest management (Angon et al., 2023), precision agriculture (Gawande et al., 2023), carbon farming (Sharma et al., 2021), and conservation agriculture (Kwiatkowski et al., 2023) stand out as the most effective practices.

## 8. Alternative farming techniques and systems

**Figure 2:**
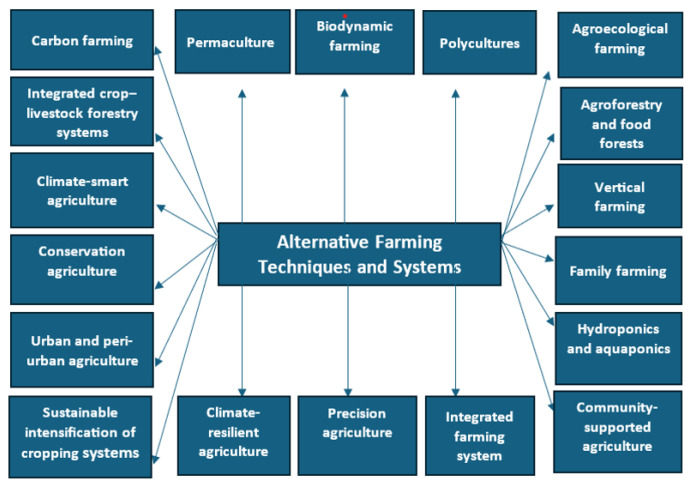
Alternative farming techniques and systems for sustainable agricultural production.

The types of alternative farming techniques are presented in Figure 2 and elaborated upon in detail in this section.

### 8.1. Permaculture

Permaculture is conceptualized as a holistic system designed to create sustainable and self-sufficient ecosystems by emulating the principles and processes of natural ecosystems; making intensive use of biological resources and polycultures; and promoting environmental stewardship, resource efficiency, resilience, and diversity. Permaculture represents a sustainable, agroecological, regenerative, and holistic approach that mirrors the diversity, stability, and resilience of natural ecosystems; enhances carbon stocks, biodiversity, and soil macronutrient and micronutrient concentrations; and is recommended for designing and managing agroecological systems to combat soil degradation, climate change, and biodiversity loss (Reiff et al., 2024). Regenerative agriculture and permaculture—holistic approaches that restore and protect natural systems while reducing dependence on external inputs—have been proposed to enhance soil health, conserve resources, and strengthen food security (McLennon et al., 2021).

Permaculture can significantly enhance biodiversity and climate resilience by connecting people, land, resources, and the environment. In a permaculture system, the integration of perennial plants—such as fruit and nut trees and various shrubs—into the landscape is fundamental, as these species contribute to long-term yields, biodiversity habitats, and overall ecosystem stability (Krebs and Bach, 2018).

### 8.2. Polycultures and sustainable cropping patterns

Polyculture involves cultivating a variety of complementary crops within the same area to create a more resilient and sustainable system, maintain ecosystem health, and enhance biodiversity and productivity. Polycultures, in which multiple plant species are cultivated simultaneously within the same field, improve land-use efficiency, enhance farmer income, and make a significant contribution to sustainable agriculture. Designing sustainable cropping patterns is a key strategy for ensuring long-term food production while maintaining ecosystem health and balance (Taheri et al., 2025). Intercropping—the practice of cultivating two or more crops in the same space at the same time—enhances agroecosystem functions and increases overall yields by maximizing production from a given area (Yu et al., 2025). Intercropping contributes to the development of more resilient agricultural systems by increasing yield and yield stability, improving resource-use efficiency, and reducing the environmental impacts of agriculture, including those from pests, diseases, and production costs (Yu et al., 2025). Additionally, intercropping improves long-term soil structure and fertility by enhancing aggregate stability (Lu et al., 2025). Cropping patterns such as multiple cropping, intercropping (mixed, row, strip, and multistory systems), sequential cropping (double, triple, relay, and cover crops), and multilevel cropping should be further developed and promoted to enhance the sustainability of agricultural systems.

### 8.3. Biodynamic farming

Biodynamic agriculture adopts an ecological and holistic approach that focuses on improving soil health, integrating plants and animals, and promoting biodiversity. It views the farm as an integrated, self-sufficient organism in which plants, animals, and soil interact in a mutually beneficial way. This system prioritizes the integration of crop and livestock production to enhance soil fertility and health; the use of fermented herbal preparations, cover crops, and crop rotations; the enhancement of biodiversity through ecosystem diversification; and the minimization of dependence on external inputs by reducing or eliminating the overuse of synthetic fertilizers and pesticides (Basooriya, 2024). Biodynamic agriculture—a synthesis of biological and dynamic practices—places particular emphasis on food quality and soil health, fostering resilient, self-sufficient, diverse, and resource-efficient agroecosystems.

### 8.4. Agroecological farming

Agroecological agriculture—which involves designing and managing ecosystems to promote sustainability, resilience, and biodiversity conservation by imitating natural processes, fostering beneficial biological interactions among agricultural components, and integrating ecological principles into farming systems (Singh et al., 2024)—is considered an effective tool for enhancing the resilience of food systems under changing climatic conditions (Moldavan et al., 2023). This system seeks to increase production capacity by reducing dependence on external inputs, emulating natural processes, fostering beneficial biological interactions and synergies among system components, and leveraging ecological processes. Resilient, productive, and site-specific agroecological practices—along with other innovative approaches—are emerging as indispensable pillars of agriculture, encompassing biodiversity-oriented, climate- and environmentally friendly, and economically sustainable production systems (Çakmakçı, 2024). Agroecological farming is founded on key principles including efficiency, biodiversity, diversification, recycling, adaptation, synergy, resilience, ecological balance, natural regulation, participation, adaptive management, and the integration of plant and animal production. Agroecological farming contributes to climate change adaptation, mitigates the adverse effects of extreme weather and poverty, improves nutrition, creates new employment opportunities for rural populations, enhances the resilience of agricultural systems (Moldavan et al., 2023), and promotes the efficient use and protection of water resources.

Agroecological agriculture supports soil health through practices such as diversified cropping systems, polycultures, crop rotation, intercropping, agroforestry, crop–livestock integration, cover cropping, and reduced tillage, while promoting biodiversity, mitigating climate change, reducing dependence on pesticides and synthetic inputs, and increasing ecosystem resilience to environmental stressors (Singh et al., 2024). Agroecological systems exhibit diversity at all levels; they create habitats for beneficial organisms such as pollinators, promote appropriate soil and water management, and support native flora and fauna to build resilient ecosystems capable of withstanding pests, diseases, and environmental fluctuations (Giagnocavo et al., 2022). Promoting agroecological agriculture requires the establishment of organized local markets; facilitating direct access of smallholder products to consumers without intermediaries; supporting sales, processing, and profitability; developing sustainable agricultural–ecological systems; and prioritizing agroecological products in public procurement policies.

### 8.5. Agroforestry and food forests

Agroforestry, which has long been practiced—particularly in regions dominated by subsistence farming—relies on the role of trees in improving soil quality, promoting sustainable land use and natural resource management, and ensuring food and nutritional security. This system has evolved into an integrated land-use model that combines forestry and agricultural practices, using trees and shrubs alongside crops or livestock to maintain forest cover and strengthen food security. Agroforestry and food forests represent sustainable farming systems that integrate agriculture and forestry, delivering both ecological and economic benefits. These approaches are increasingly important in regions vulnerable to climate change (Jiang et al., 2022). Preserving the multiple ecological functions of forests and maintaining continuous forest cover through close-to-nature management practices make forest restoration essential (Kumar et al., 2020). Sustainable agroforestry—an innovative approach that integrates tree production with agriculture—offers multiple economic benefits, including reduced production costs and greenhouse gas emissions, higher yields, enhanced carbon sequestration, diversified income streams, and improved ecosystem services and resilience (Fatima et al., 2024). Agroforestry has significant potential to enhance food security by increasing crop yields and soil fertility, strengthening resilience, delivering key ecosystem services, improving water quality, and combating land degradation. As a climate-resilient farming system, agroforestry delivers ecological and agricultural benefits such as soil moisture conservation, erosion control, improved soil microbial activity, biodiversity conservation, increased income and crop diversity, degraded soil restoration, and multiple cropping (Meetei et al., 2023).

Forests and trees, which are vital sources of food, energy, fodder, and medicinal plants, can adapt to climate change and other constraints; regulate local climates; sustain water supplies; provide shade; protect coastal areas; preserve watersheds; and enhance the resilience of agricultural systems (Meybeck et al., 2024). Trees provide additional income to farmers; protect crops from wind, heavy rain, temperature fluctuations, and soil moisture loss; improve soil stability; reduce nutrient runoff; promote healthy plant growth; and maintain soil fertility. Food forests or forest gardens are typically established through permaculture systems, forming multilayered canopies that include perennial plants, medium and short fruit and nut trees, followed by layers of fruit bushes, natural specialty crops, climbers, perennial medicinal herbs, and ground-level vegetables.

Studies have demonstrated that integrating trees with crops and livestock in agroforestry systems supports water conservation, habitat restoration, ecological balance, and sustainability (Fatima et al., 2024). Such systems contribute to reducing global warming, producing climate-adapted food, enhancing ecosystem services, and conserving biodiversity and natural habitats (Quandt et al., 2023); increasing soil nitrogen and carbon; improving ecological stability and soil microbial communities (Guillot et al., 2019); regulating water through improved soil properties and enhanced soil-water quality and availability (Awazi et al., 2025); conserving land and managing land-use change; designing buffer strips, reconnecting rivers to floodplains, improving urban water systems, and restoring coastal habitats (Delgado-Lemus and Moreno-Calles, 2022); creating and maintaining wetlands, ponds, watersheds, and forests; providing economically viable and sustainable solutions for rural communities; mitigating climate change (Tsiakiris et al., 2024); and improving food and nutritional security while enhancing soil moisture and agricultural performance (Sahoo and Wani, 2020).

### 8.6. Integrated crop-livestock–forestry systems

In addition, as a response to the challenges of modern agriculture, integrated crop-livestock-forestry systems have been proposed as a nature-based solution that incorporates natural processes and ecosystem services into agriculture while delivering environmental, social, and economic benefits (Carvalho et al., 2024). Integrated crop-livestock–forestry systems establish a vital link between agriculture and nature, ensuring the viability of production systems and enhancing food security while positively influencing microclimate regulation, water and nutrient cycling, and biodiversity (Lemaire et al., 2023). Nature-based solutions simultaneously promote human well-being and biodiversity; contribute to the conservation of soil and natural resources; and encompass diversification, conservation practices, soil and grazing management, and crop rotation as integral components of sustainable food production (Carvalho et al., 2024). Integrated crop-livestock-forestry systems can play a key role in developing more sustainable and resilient farming systems while maintaining profitability, productivity, and food security (Garrett et al., 2020). The benefits of integrated systems, tailored to specific local conditions, include greater system flexibility, reduced dependence on external inputs, and multifunctionality that supports multiple ecosystem services. Integrated crop-livestock–forestry systems emulate the beneficial functions of natural ecosystems, ensuring effective resource management to maintain and enhance soil fertility while supporting the conservation of ecosystems and biodiversity.

### 8.7. Carbon farming

Carbon farming, a sustainable land management system that balances climate change mitigation with agricultural production, is gaining significant attention as an approach that enables environmentally friendly food and commodity production while reducing farm-related greenhouse gas emissions (Sharma et al., 2021; Kwiatkowski et al., 2023). The main objective of this system is to enhance agricultural productivity and reduce greenhouse gas emissions by accelerating the conversion of atmospheric CO_2_ into plant biomass and soil organic matter. In particular, agroforestry, which integrates crop and livestock production with woody vegetation, plays a crucial role in mitigating climate change, enhancing drought resilience, and improving farm productivity and food security (Paul et al., 2023). For effective carbon farming, recommended methods include rewetting and restoring peatlands, establishing agroforestry systems, managing livestock and manure, and preserving soil organic carbon (Sharma et al., 2024). On farms, greenhouse gas emissions can be reduced through improved product and waste management, optimized energy and fertilizer use, and sustainable feeding practices. Through strategies such as efficient fertilizer and biofertilizer use, cover cropping, land-use change, mulching, improved crop rotations, agroforestry integration, and optimization of production type and location, carbon farming not only mitigates climate change but also supports biodiversity conservation and agricultural sustainability (Raina et al., 2024; Sharma et al., 2024).

### 8.8. Climate-smart agriculture

Climate-smart agriculture (CSA) is a strategy designed to transform and reorient agricultural systems to enhance food security by sustainably improving resilience, resource-use efficiency, and productivity under the impacts of climate change. Given the limited agricultural land, water scarcity, climate change, and constantly shifting environmental conditions, ensuring food security requires the integration of smart and PA technologies into the agricultural sector. These include the Internet of Things (IoT), blockchain, sensors, robotics, artificial intelligence (AI), machine learning (ML), deep learning, big data analytics, and smart supply chain systems (Çakmakçı and Çakmakçı, 2023b; Jararweh et al., 2023). Smart agriculture encompasses applications such as soil and field monitoring, precision irrigation, smart machinery, remote sensing and satellite monitoring, variable-rate seeding, smart greenhouses, disease diagnostics, autonomous robotics, and mobile-based management tools. Collectively, these technologies form an integrated approach to manage climate risks and reorient agriculture toward the sustainable development goals (SDGs) (Çakmakçı and Çakmakçı, 2022; Waaswa et al., 2022). Emerging smart technological interventions help mitigate the negative environmental and ecological impacts of farming and “optimize resource use and efficiency, supporting on-farm productivity and the broader goals of sustainable agriculture” (Gamage et al., 2024). Climate-resilient agriculture has been reported to enhance productivity, resilience, food security, and water management by integrating traditional knowledge with modern technologies (Singha et al., 2024; Sahoo et al., 2025).

Smart farming enhances the resilience of agricultural enterprises by increasing production, improving water efficiency, providing real-time monitoring and data-driven insights, reducing operational costs, and enhancing profitability. It also improves production quality, enables accurate farm and field assessments, advances animal husbandry, minimizes waste, optimizes resource use, and strengthens overall sustainability (Jararweh et al., 2023). Integrating CSA technologies into food production systems and supply chains enhances climate resilience (Usigbe et al., 2024). CSA encompasses a range of practices—including crop diversification and rotation, intercropping, conservation agriculture, and integrated crop-livestock systems—that enhance productivity and yield, strengthen resilience to climate impacts, and reduce greenhouse gas emissions. Common climate-smart technologies include improved forage production, manure and soil fertility management, nutrient and biodiversity optimization, controlled irrigation, reduced nitrogen and fertilizer inputs, agroforestry, minimum tillage, and crop rotation. The application of smart technologies in indoor cultivation systems contributes to developing a more resilient and sustainable agricultural sector while promoting resource efficiency, environmental protection, and improved crop yields.

Due to climate change, agricultural production in arid and semiarid regions is declining as a result of salinity stress and inadequate irrigation, while traditional agricultural systems fail to support sustainable production (Mukhopadhyay et al., 2021). Climate-smart agriculture supports efficient water management and food security while enabling both climate change mitigation and adaptation. It offers the potential to meet global food demands by improving productivity and resilience under climate variability through practices such as agroforestry, intercropping, mixed cropping, terracing, mulching, perennial plantations, minimum tillage, and the use of cover crops. By integrating multiple techniques that promote sustainable productivity with lower environmental impact (Kichamu-Wachira et al., 2021), CSA strengthens the resilience of agrifood systems to climate change, enhances production, and reduces farm-related greenhouse gas emissions (Maraseni et al., 2021). CSA practices have been shown to increase maize yields while reducing carbon footprints, making them both promising and essential for food security and climate mitigation (Feng et al., 2023).

As a subset of CSA strategies, CRA focuses on water and nutrient management, technological and knowledge management practices, and socioeconomic resilience to sustainably increase productivity and reduce climate-related risks—thereby supporting farm income, food security, and rural development (Kundu et al., 2024). Adaptation and resilience to climate change—defined as adjustments in ecological, social, or economic systems in response to actual or expected climatic stimuli and their impacts—are of vital importance.

### 8.9. Climate-resilient agriculture

Processes such as climate change, drought, flooding, temperature extremes, salinity, and other biotic factors have significant and direct impacts on crop yields. Building climate-resilient agricultural systems and communities requires adopting targeted, equitable, locally integrated, accessible, and sustainable practices and technologies that address the needs of all vulnerable social groups (Nepomoceno and Carniatto, 2023). Climate-resilient agriculture (CRA) encompasses sustainable land and water management practices that enhance resilience to climate change and improve resource-use efficiency by adapting modern techniques to agriculture, promoting sustainable food supply and security, increasing yield potential, and reducing greenhouse gas emissions (FAO, 2018^4^; Karri and Nalluri, 2024). For climate-resilient agriculture, the adoption of smart technologies and adaptive practices—such as climate-resilient cropping systems, efficient water management, micro-irrigation, integrated nutrient management, balanced fertilization, cover cropping, soil recarbonization, crop residue retention, rotation, zero-tillage systems, mulching, beneficial stress-tolerant microbes, integrated farming, and region-specific nutrient management—is strongly recommended (Keshavarz and Sharafi, 2023; Karri and Nalluri, 2024). To achieve a sustainable food system, growers must progressively adapt to climate-resilient farming through continuous technological advancements (Karri and Nalluri, 2024). Climate-adaptive farming and the adoption of climate-resilient agricultural techniques offer viable solutions to the challenges posed by unpredictable climate change.

### 8.10. Precision agriculture

Precision agriculture (PA)—a transformative approach that enhances farming efficiency—uses data-driven methods and advanced technologies to promote sustainability, minimize environmental impact and resource waste, and maximize output with fewer inputs. PA incorporates technologies that enable informed decision-making in agricultural activities, optimize resource use, enhance productivity, foster sustainable practices, and minimize losses and environmental impacts (Oztuna Taner, 2024). PA optimizes and reduces the application of inputs such as water and fertilizers to maximize yields. It represents an important farm management system that integrates ecological principles with biodiversity management techniques and technologies. PA technologies not only generate substantial savings in key productivity factors but also promote ecological and economic sustainability and can be readily implemented on small-scale farms (Loures et al., 2020).

### 8.11. Integrated farming systems

An integrated farming system (IFS) is a holistic agricultural approach that transforms small and marginal farms into environmentally friendly, productive, profitable, and biodiversity-rich systems that are sustainable, climate-resilient, and capable of ensuring food and nutritional security through diverse ecosystem services (Kumar et al., 2018; Bhagat et al., 2024). As an environmentally friendly, resource-based, and livestock-integrated holistic model, an IFS utilizes low inputs and farm byproducts, enhances nutrient-use efficiency, improves soil fertility, and effectively meets the needs of smallholder farmers (Paramesh et al., 2021). Designed as a biologically integrated system, an IFS aims to incorporate natural resources into farming activities, minimize external inputs, ensure the sustainable production of high-quality food, and reduce environmental pollution (Fatima et al., 2023). IFSs maintain soil fertility through efficient nutrient recycling, reducing the need for chemical fertilizers (Kumar et al., 2018). They also preserve soil microbial biodiversity and natural habitats (Centeno- Alvarado et al., 2023), improve habitat quality and pollination services, and enhance system profitability and nutritional security (Palsaniya et al., 2022). Location-specific and well-planned IFSs conserve resources through complementarities among crops (agriculture, horticulture, agroforestry) and livestock, thereby contributing to economic stability and food security (Kumar et al., 2018). IFS provide protection against yield loss and serve as agroecological models that reduce the effects and risks of climate change (Bhagat et al., 2024; Mishra et al., 2024). Integrated farming systems offer multiple advantages, including generating regular income from diverse components throughout the year, conserving soil and water, recycling agricultural residues, and reducing disease incidence, crop losses, and climatic risks (Meena et al., 2024).

### 8.12. Conservation agriculture

Conservation agriculture (CA) is an advanced alternative to conventional production and management strategies that integrates agricultural, environmental, and economic dimensions to protect farming systems and conserve natural resources (Kassam et al., 2019). The core practices of CA include maintaining permanent soil organic cover using residues and/or cover crops, implementing conservation tillage, and adopting diversified cropping through intercropping and rotations involving at least three species, including legumes (Tian et al., 2024). CA is a fundamental approach that enhances the physicochemical and biological properties of the soil, thereby improving ecosystem health and sustainability. In addition to its climate change mitigation and environmental benefits, CA reduces production costs, increases irrigation efficiency, promotes crop diversity, and enhances the physicochemical and biological quality of the soil. CA enhances ecosystem health, resilience, and sustainability within cropping systems, ensuring long-term food security and reducing the adverse impacts of climate change on agricultural production. CA enhances soil organic matter through conservation tillage; suppresses weeds; improves water productivity, microbial diversity and activity, and soil structure and quality; and increases input-use efficiency (Kumar et al., 2023). The benefits of CA—which incorporates minimum tillage, permanent soil cover, and crop rotations—include enhanced drought resistance, improved soil fertility and water infiltration, better water harvesting, higher yields, and strengthened food security (Sharma et al., 2024). CA practices protect soil and water resources, minimize production costs, enhance water-holding capacity and infiltration, improve nutrient and water-use efficiency, and support climate-resilient agriculture by significantly contributing to ecosystem health (Farooq et al., 2024).

### 8.13. Urban and peri-urban agriculture

The growing threats of climate change and environmental challenges, including natural disasters and resource scarcity, have made resilience a central theme in future urban planning. At the same time, increasing efforts are being directed toward developing sustainable and livable cities that can withstand and recover from environmental shocks (Kahachi et al., 2024). Urban areas face multiple risks—including natural disasters, earthquakes, excessive land consumption, resource depletion, water scarcity, cropland loss, poverty, and food insecurity—all of which are exacerbated by climate change, rapid urbanization, population growth, and biodiversity loss (Çakmakçı et al., 2023). Uncontrolled urban sprawl threatens natural and seminatural habitats and ecosystem services, raising concerns about the economic, environmental, and social sustainability of food supply chains—from production to processing, distribution, and urban waste management (Sgroi and Musso, 2022). Consequently, developing urban and peri-urban food systems—through urban agriculture (UA), green infrastructure, and urban gardens—has become increasingly essential. UA encompasses a wide range of applications, both commercial and nonprofit, including indoor farming, vertical farming, hydroponics, aeroponics, aquaponics, soilless cultivation, precision agriculture (PA), and the use of remote sensing technologies (Çakmakçı et al., 2023; Qu et al., 2024).

Short supply chains are a crucial component of integrated food system management, fostering agricultural vitality and supporting sustainable markets. Urban markets, particularly in small and medium-sized enterprises, enable smallholder farmers to sell their products directly to consumers. Strengthening food systems to encompass urban, peri-urban, and rural areas is essential for improving food production, storage, transportation, distribution, and marketing; reducing losses; and preventing or reusing food waste. UA contributes to sustainable food systems by reducing dependence on mineral fertilizers through the recycling of urban waste as organic fertilizer. UA, which demonstrates significant potential in addressing urban environmental challenges, supports the circular economy by enabling the composting of organic waste. UA is a vital strategy for enhancing the resilience of urban food supply systems, reducing poverty, and increasing employment opportunities. It helps address urban challenges and vulnerabilities by utilizing unused urban spaces, contributing to microclimate regulation and rainwater management, improving air quality, mitigating the urban heat island effect, enhancing nutrition, and reducing environmental degradation (Sousa et al., 2024; Qu et al., 2024).

UA, which primarily utilizes local resources to meet the needs of local populations, encompasses various forms, including home and community gardening, shared and micro-gardens, commercial crop production, indoor farming, and institutional food cultivation (FAO, 2022b). To transform food sources and systems toward urban proximity and sustainable development, creative solutions such as backyard farms, community gardens, urban greenhouses, rooftop farms, indoor hydroponic farms, and vertical farming towers are essential. UA plays a crucial role in achieving food security by increasing local food production and enhancing urban biodiversity (Anwar et al., 2023). Indigenous peoples, whose regenerative food system knowledge and practices are grounded in diversity, resilience, and adaptability, play a vital role in conserving biodiversity, reducing environmental degradation, and providing sustainable food for their communities (Knorr and Augustin, 2025). Indeed, local communities’ specific knowledge of the use and management of natural resources can substantially contribute to the sustainability of food systems (Parodi et al., 2018). Indigenous food systems represent desirable models for developing future sustainable and resilient food systems, as they are inherently diverse, adaptable, self-sufficient, efficient, long-lasting, circular, and closely aligned with the natural environment (Kimmerer and Artelle, 2024; Knorr and Augustin, 2025).

### 8.14. Vertical farming

Vertical farming is an innovative and environmentally friendly agricultural method suited to urban areas. It maximizes land productivity by growing crops in vertically stacked layers rather than on the ground, unlike traditional farming systems. Vertical urban farming—employing indoor techniques such as hydroponics, aeroponics, and aquaponics—offers a sustainable solution for ensuring urban food security by reducing dependence on external inputs and resources.

### 8.15. Hydroponics and aquaponics

Hydroponic and aquaponic systems enable plant cultivation in nutrient-enriched, soil-free environments. In hydroponic systems, plant roots are placed either directly in mineral nutrient solutions or in inert substrates such as gravel or perlite, while aquaponic systems integrate hydroponic crop cultivation with aquatic animal production. Compared with traditional cultivation, both systems enable year-round production, reduce pest and disease risks, use substantially less water, and are suitable for urban and arid regions, though they require considerable investment and technical expertise. Hydroponics has emerged as an efficient and resilient plant-based food production system, offering a sustainable solution for urban environments (Sousa et al., 2024). Characterized by the efficient use of vertical space within controlled environments, hydroponic systems integrated into vertical UA are recognized as resilient and sustainable solutions for increasing urban food production (Romeo et al., 2018).

Through precise control of factors such as nutrient concentration, pH, oxygen, and temperature, hydroponic systems create optimal conditions for rapid plant growth while requiring significantly less space than conventional systems. The most common hydroponic systems include wick systems, deep-water culture systems, nutrient film techniques, aeroponics, drip systems, aquaponics, and flood-and-drain setups (Sousa et al., 2024). A key feature of these systems is the inclusion of a reservoir and aerator that maintain and circulate the nutrient solution. Hydroponic systems support a wide range of crops, including fruiting plants (e.g., tomatoes, strawberries, dwarf citrus trees), leafy and stem vegetables (e.g., spinach, lettuce, chicory, peppers), medicinal and aromatic herbs (e.g., basil, coriander, cilantro, mint), microgreens (e.g., radish, beet, broccoli), and protein-rich aquatic or grass species such as wheatgrass. Hydroponically grown vegetables, which require less time and water, are noted for their superior flavor, sustainability, and nutrient-dense profiles. Short supply chains for fresh produce play a crucial role in reducing vulnerabilities linked to supply risks, shortages, long transportation distances, and fossil fuel dependence.

Systems equipped with advanced climate control technologies—including precision automation, LED lighting, and artificial intelligence (AI)—optimize the growing process, reduce sensitivity to weather extremes, and lower maintenance and production costs (O’Sullivan et al., 2019). Rooftop vegetable gardens reduce carbon emissions and urban heat island effects, mitigate noise, improve air quality, enhance local fresh produce supply, and contribute to urban livability, food security, and safety (Sousa et al., 2024). Local vertical hydroponic production demonstrates strong economic potential, enhancing both food security and the sustainability of urban areas (Gumisiriza et al., 2022).

### 8.16. Family farming

The challenges posed by climate change have intensified the search for effective and sustainable adaptation strategies in rural areas. Family farming, which involves thriving on family-sourced land, labor and capital, plays a crucial role in promoting sustainability, enhancing food security, and strengthening the resilience of food systems (Little et al., 2023; Chao, 2024). Although family farms often exhibit sustainability and resilience to climate change, they remain vulnerable to economic, environmental, and market shocks. Family farms employ multiple adaptive strategies, including crop diversification, altered planting schedules, cultivation of high-value crops and fruit trees, and integration of poultry and livestock production. Sustainable and resilient family farms contribute to regional development and emerge from the mutual interaction of socioeconomic and environmental factors (Nepomoceno and Carniatto, 2023). Advancing family farming requires the expansion of environmentally sound and productive practices, diversified farming approaches, and ecosystem services such as soil conservation, biological pest control, biofertilizer application, water-use efficiency, diversified production methods, and rural tourism. As the foundation of more effective and sustainable agrifood systems (Worstell and Green, 2017), multifunctional family farms are essential not only for ensuring food security and nutrition but also for managing natural resources, protecting the environment, and supporting sustainable livelihoods (Chao, 2024).

Family farming practices contribute to maintaining soil health and biodiversity, adapting to climate change, and mitigating its adverse impacts (Wezel et al., 2020). They also support risk management in agriculture (Nepomoceno and Carniatto, 2023); enhance nutrient-rich food production and ecosystem services and improve nutrition and health outcomes (Borychowski et al., 2020); strengthen control over food production, processing, and distribution (Lu et al., 2022); and promote sustainable agriculture and food security (de la Peña García et al., 2020; Arabska, 2021). Grounded in local knowledge and environmentally friendly techniques, family farming is recognized as an integral component of regional, sustainable, and climate-resilient food systems.

### 8.17. Community-supported agriculture

Community-supported agriculture (CSA) represents a resilient, environmentally responsible, and community-centered food system that establishes a direct link between farmers and consumers. In this model, community members actively support farm operations through financial contributions, labor, or resources, thereby reconnecting people with food spatially, economically, and socially while enhancing overall food system sustainability (Birtalan et al., 2020; Tay et al., 2024). CSA-based horticultural production enhances regional resilience and holds strong potential to improve ecological and socioeconomic sustainability. Furthermore, urban and rural planning can play key roles in fostering equitable and sustainable transitions (Cristiano, 2021). This agricultural model represents a local alternative food system that prioritizes social and ecological values over profit maximization, with farmers and consumers sharing both the costs and outputs of production (Bonfert, 2022). CSAs constitute a dynamic and adaptable form of food production arising from collaborative partnerships between producers and consumers and grounded in sustainable soil management, organic practices, and biodiversity conservation.

CSA is evolving into a direct-marketing model in which a community of individuals collectively supports farm operations by sharing labor, costs, risks, and benefits. CSAs form part of broader alternative food networks that reconnect consumers with nature, alongside initiatives such as farmers’ markets and urban community gardens. Beyond financial returns, CSA participation yields numerous social benefits, including enhanced autonomy, the fulfillment of meaningful labor, the satisfaction of nourishing communities with healthy foods, and reduced financial risk and uncertainty (Paul, 2019). CSAs—particularly those that facilitate partnerships between highly educated consumers and marginalized or resource-poor farmers—play a crucial role in enhancing food security, promoting ecological conservation, and supporting rural development. CSA initiatives have been shown to foster holistic approaches that advance sustainable community development, mitigate environmental degradation, and contribute to more equitable food systems (Tay et al., 2024). Ultimately, CSAs promote sustainable agricultural practices, strengthen farmer–consumer and human–nature relationships, and generate tangible benefits for the well-being of society as a whole.

### 8.18. Sustainable intensification of cropping systems

Sustainable intensification practices are strategic approaches designed to enhance farm productivity, food security, soil health, agricultural biodiversity, and ecological sustainability. Sustainable intensification of cropping systems—built upon crop rotation, intercropping with legumes, and the integration of agroforestry practices—is vital for maintaining biodiversity, improving soil health and fertility, and enhancing resource-use efficiency (Yadav et al., 2024). The concept of cropping system intensification is emerging as a key strategy to increase crop yields and enhance critical soil health indicators, including soil structure, fertility, nutrient cycling, and microbial and biological activity. Integrating these strategies into farming systems can simultaneously enhance productivity, strengthen food security, promote environmental sustainability, and build resilience to climate change.

Ecological intensification and resource-efficient production systems are increasingly recognized as essential pathways for achieving agricultural productivity and ensuring food and environmental sustainability under changing climatic conditions (Kumar et al., 2021). Amid challenges such as nutrient depletion, declining productivity and profitability, falling groundwater levels, and unsustainable agricultural practices, cropping system intensification has emerged as a means to enhance the productivity of farming systems—particularly cereal-based systems. These integrated, sustainably intensified cropping systems reduce energy inputs, exhibit resilience to environmental stresses such as drought, and contribute to climate change mitigation, ecosystem resilience, and sustainable agricultural development.

The intensification of cropping systems—particularly through legume diversification with crops such as rice and maize—enhances the sustainability of agricultural systems under climatic and environmental stresses (Yadav et al., 2024). Cropping system intensification has yielded promising outcomes in improving soil health, crop yield, and overall system productivity across diverse regions and cropping patterns (Kumar et al., 2021). Such systems play a vital role in reducing resource and water use per unit of output and in mitigating environmental degradation. Integrating legumes into cropping systems reduces dependence on external fertilizers by enhancing nitrogen fixation and phosphorus utilization and is particularly effective in improving soil health and ecological sustainability indicators.

## 9. Development trends and emerging innovations in future food systems and green technologies

Green technologies are increasingly central to achieving long-term sustainability in food production. These include PA, remote sensing, vertical farming, hydroponics, and aeroponics in UA (Anwar et al., 2023; Çakmakçı and Çakmakçı, 2023a); data integration, agricultural robotics, climate forecasting, and traceability systems in digital agriculture (Çakmakçı and Çakmakçı, 2022, Çakmakçı and Çakmakçı 2023b; Balasundram et al., 2023); genome editing, synthetic biology, and novel nitrogen-fixing crops in genetic technologies (Qu et al., 2024; Wang and Zhang, 2024); microalgae, cyanobacteria, seaweed, and cellular agriculture proteins in production intensification (Blikra et al., 2021; Çakmakçı et al., 2024); and plant-based proteins, microbial and cell-based foods, and cultured meat as alternative protein sources (Qin et al., 2022; Lee et al., 2023). Although still in its early stages, cell-based cultured meat, produced from laboratory-grown animal cells, can be tailored to meet specific nutritional needs while reducing pressure on land, the environment, and natural resources compared with conventional meat production. The potential of alternative and novel foods—including seaweed, algae, and insects—to enhance nutritional quality and reduce environmental impacts warrants further exploration (Sperling et al., 2022).

Although research and development in the food industry have progressed relatively slowly, recent advances in integrated synthetic biology and fermentation technologies—which involve the targeted design, transformation, and even resynthesis of living systems (Huang and Nikel, 2019)—offer new potential for protein biosynthesis and cultured meat cell cultivation for food and feed (Asseng et al., 2021). These innovations can expand production capacity, enable the development of new bioengineered species and technologies, and contribute to reductions in pollution and energy use (Shi et al., 2022; Çakmakçı et al., 2024). The rapid microbial production of food components such as proteins, vitamins, and starch through synthetic biology innovations represents a promising alternative for the food industry (Çakmakçı et al., 2024). Synthetic biology, a multidisciplinary field within biotechnology, aims to harness living systems for research, product development, and the synthesis of novel biological components. Its applications range from the design of microbial cell factories to genome restructuring and DNA synthesis and reconstruction (Hayakawa et al., 2024).

Synthetic biology techniques enable the production of functional and personalized foods, the development of novel high value-added food additives, the conversion of renewable raw materials into food ingredients and chemicals, and the artificial biosynthesis of food resources (Wang and Zhang, 2024). Synthetic biology offers innovative solutions to major challenges in sustainable agriculture, including enhancing nutritional quality and photosynthetic efficiency, increasing secondary metabolite production, and improving crop performance and stress tolerance (Hayakawa et al., 2024). Interest in functional food research has recently increased, particularly in the recovery of bioactive compounds from diverse sources such as microalgae and macroalgae, animal tissues, plants, herbs, nuts, mushrooms, fortified foods, enriched dairy products, and even food industry by-products.

Plant synthetic biology has emerged as a promising approach to enhance resource-use efficiency, reduce dependence on external inputs, and strengthen food security and resilience to climate change. It represents a sustainable, interdisciplinary approach that integrates biology, engineering, and computational science to improve agricultural practices, biofortify crops, increase yields, enhance nutritional content, and reprogram biological systems for greater efficiency (Wang and Demirer, 2023). In agriculture, synthetic biology provides cutting-edge tools to increase food production, enhance nutritional quality, and advance sustainability. Applications include photosynthetic optimization, nitrogen fixation, stress and pathogen resistance, drought-tolerant crop development, nutrient-use efficiency, and biofortification. Emerging frontiers encompass synthetic genetic circuits, engineered plant genomes, microbiome editing, biosensors, bioactuators, microbial engineering, synthetic organelles, cellular compartmentalization, and the integration of artificial intelligence (AI) (Wang et al., 2022; Ye et al., 2024; Karataş and Ayaz, 2025; Zhang et al., 2025). Synthetic biology enables the creation of high-yield, nutrient-dense, and stress-tolerant products, including novel biological elements such as enzymes, engineered cells, and genetic circuits, and plays a pivotal role across the food, biofuel, therapeutic, and cosmetics industries (Zhang et al., 2025).

Traditional food processing and production methods are undergoing profound transformation as they increasingly confront constraints related to resource availability, energy consumption, and environmental degradation. Interdisciplinary innovation is driving the food industry toward comprehensive nutrition, high-tech integration, and intelligent production systems (Wang and Zhang, 2024). Concurrently, advances in protein engineering, fermentation, enzymology, cellular and genetic engineering, molecular food science, and low-carbon production technologies are emerging as key enablers of sustainable development (Hassoun et al., 2022). Synthetic biology, integrating engineering, life sciences, and information technologies, is expected to enhance the production capacity of the food industry and enable the development of novel microbial strains and biotechnological platforms (Shi et al., 2022).

Synthetic biology plays a pivotal role across diverse applications, including food and ingredient development, plant-derived products, microbial pharmaceuticals, nutraceuticals, probiotics, food preservatives, personal care formulations, flavors and fragrances, green chemicals, bioactive metabolites, and industrial enzymes. The advancement of microbial synthetic biology has expanded the production of high-value food products, bioenergy, and materials from renewable plant, fungal, and bacterial resources, while also supporting waste valorization and bioremediation. Through microbial technologies and cell factories, researchers have achieved modification of plant nutrient pathways and photosynthetic processes, along with the biosynthesis of plant-derived secondary metabolites, pharmaceuticals, pigments, and flavor compounds. Recent advances in synthetic approaches have enhanced plant nutritional composition by increasing anthocyanin, carotenoid, and lycopene levels in crops such as tomatoes (Li et al., 2018; Wang et al., 2023). In parallel, breakthroughs in rubisco engineering (Mao et al., 2023), the introduction of photorespiratory bypasses into chloroplasts (Smith et al., 2023), and CO_2_-concentrating mechanisms in plants (Rottet et al., 2021), together with efforts to introduce C_4_ pathways into C_3_ species (Furbank et al., 2023), design synthetic carbon-fixation pathways (Miller et al., 2020), and regulate photosynthetic light reactions (Slattery and Ort, 2021), have provided promising directions for improving photosynthetic efficiency.

Elucidating the mechanisms of plant metabolite biosynthesis through the modification of secondary metabolic pathways has enabled the production of valuable compounds in medicinal plants. In parallel, the use of microorganisms in fermentation has transformed it into a major industrial process for producing chemicals, pharmaceuticals, enzymes, proteins, and biofuels. Microbial cell factories have emerged as promising platforms owing to their high productivity, sustainability, and controllability, particularly for the biosynthesis of phenolic compounds, anthocyanins, and glycoside sweeteners (Hayakawa et al., 2024). In particular, microbe-based precision fermentation has become a powerful tool for achieving high-quality, consistent, and scalable food production (Shi et al., 2022). Synthetic biology, with extensive applications across food, biofuel, pharmaceutical, metabolomics, and bioremediation industries, is also gaining increasing attention in plant science, particularly for metabolite production, biosensor design, plant development, and the enhancement of stress tolerance (Gupta et al., 2021).

The concept of a food cell factory—a system that produces artificial or cultured foods from renewable biomass feedstocks—representing a strategic trend in modern food technology, aiming to create safer, more nutritious, and environmentally sustainable food production models (Karim et al., 2020). With the integration of artificial intelligence (AI), synthetic biology, and industrial-scale biomanufacturing—including large-volume microbial cell cultivation, additive production in bioreactors, and advanced fermentation and biosynthesis technologies—the future of food is expected to be more nutritious, safer, flavorful, and sustainable (Yang et al., 2022; Wang and Zhang, 2024).

## 10. Digital twins in agriculture

In recent years, interest in integrating advanced technologies into agricultural practices has increased markedly, and the interconnections among precision agriculture (PA), smart farming, machine learning (ML), and the Internet of Things (IoT) have become increasingly interconnected and synergistic. Emerging PA technologies are profoundly transforming the agricultural sector by optimizing productivity and minimizing the ecological footprint of farming, thereby directly contributing to the objectives of sustainable agriculture (Gamage et al., 2024). Enhancing the productivity and sustainability of food production and supply requires the development of next-generation smart agricultural systems, grounded in digital intelligence, through the integration of digital technologies and the efficient collection, management, sharing, and use of agricultural and environmental data from multiple sources (Gebresenbet et al., 2023). Empirical research further demonstrates a strong and complementary relationship between PA, ML, and IoT frameworks (Gamage et al., 2024). By leveraging data-driven technologies, digital agriculture has the potential to enhance efficiency, productivity, and food security, while simultaneously protecting soil health, biodiversity, and human well-being and advancing sustainability across global food systems (MacPherson et al., 2022).

Recent progress and rapid expansion in digital agriculture are primarily driven by breakthroughs in IoT and artificial intelligence (AI) technologies (Cravero and Sepúlveda, 2021; Purcell et al., 2023). Recently, agriculture has become an important application and transfer area for AI as part of the transformation process (Kisliuk et al., 2023). Digital Twin (DT) technology enhances the efficient utilization of resources and infrastructure while promoting environmental, economic, and social sustainability. DTs enable real-time monitoring of livestock activities, optimization of crop inputs, and reduction of emissions to air, soil, and water (Lovarelli et al., 2020; van der Burg et al., 2021). DTs also facilitate supply chain transparency and traceability, enabling proactive actions that restore and strengthen social and environmental sustainability objectives and improve overall agricultural productivity (Lovarelli et al., 2020; Purcell et al., 2023). The integration of AI with advanced remote sensing technologies has significant potential across multiple agricultural processes, including soil tillage and seeding, weed detection and control, biotic and abiotic stress monitoring, yield prediction and estimation, harvesting, organic farming, and livestock management. These systems can also establish economically viable and environmentally friendly crop-stress prediction frameworks (Çakmakçı and Çakmakçı, 2023b; Kisliuk et al., 2023). For instance, AI-driven algorithms have been shown to improve milk production efficiency through optimized monitoring and management systems (Oztuna Taner and Çolak, 2024). AI has the potential to enable novel processes and technologies, reduce labor requirements, and support decision-making systems that enhance the economic, ecological, and social performance of food production systems. Achieving resilient agricultural production systems capable of absorbing shocks and restoring functionality is increasingly feasible through innovative, AI-supported technologies (Usigbe et al., 2024).

**Figure 3:**
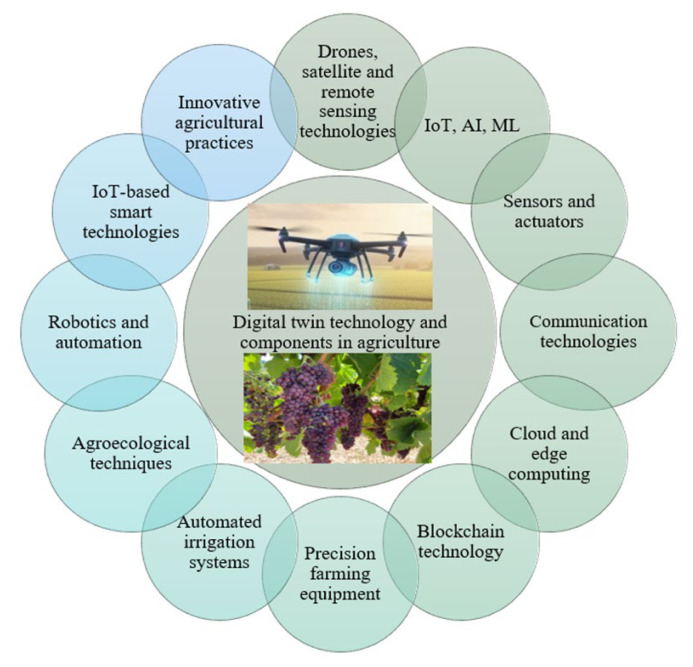
Digital twin architecture, components, and applications in agriculture.

DT technology, which integrates computation, communication, and control systems to create intelligent, data-driven environments, primarily focuses on efficient resource utilization, growth forecasting, and waste minimization (Laryukhin et al., 2019; Skobelev et al., 2020). DTs have demonstrated success in diverse agricultural applications, including yield forecasting, crop monitoring, machine calibration, and the optimization of irrigation schedules and growth conditions (Tsolakis et al., 2019; Angin et al., 2020; Akroyd et al., 2022). Figure 3 illustrates the conceptual framework and operational flow of DT applications in agriculture, emphasizing the integration of data acquisition, modeling, and real-time feedback mechanisms. The integration of machine learning (ML) and deep learning (DL) techniques into DT frameworks has advanced the development of sustainable and adaptive agricultural systems, particularly through plant stress detection, prediction, and management (Ali et al., 2024). ML-based applications efficiently optimize food, agricultural, and environmental systems to enhance overall sustainability. They reduce disease and weed management costs and chemical dependency, increase crop yields, and contribute to human and environmental health (Usigbe et al., 2024). Smart technologies, including the IoT, cloud computing technology (CCT), blockchain technology (BCT), and wireless sensor networks (WSNs), have significantly contributed to sustainable agriculture by enabling data-driven smart farming systems, precision irrigation, and integrated resource management (Lytos et al., 2020; Çakmakçı and Çakmakçı, 2023b). Within agricultural contexts, IoT systems facilitate process automation, provide real-time data for informed decision-making, enable efficient management of inputs such as water, fertilizers, and pesticides, and consequently improve yields while reducing waste and environmental impact.

Digital technologies offer vast opportunities to build an efficient, climate-resilient agricultural sector by optimizing resource management, production, and economic performance. However, if misapplied or unequally distributed, these technologies may generate unintended, far-reaching socioecological consequences. Potential adverse outcomes include accelerated wealth inequality and market centralization, the displacement of small-scale family farms, the monopolization of production systems, reduced systemic robustness due to centralized architectures, heightened external dependencies, and insufficient ecological integration in decision-making (Khan et al., 2021; Smith et al., 2021). Therefore, it is essential to evaluate digital agricultural models under adverse conditions, ensure feasible investment costs and technological robustness, and develop validated, scalable, and modular applications that explicitly incorporate social and ecological considerations (Vecchio et al., 2020; Alelyani, 2021; Mahmud et al., 2021).

## 11. Future directions, perspectives, and policy proposals

The in-situ reuse of recycled biomass as a soil conditioner and plant biostimulant within organic nutrient cycles, coupled with the enhancement of soil biological activity through microbial fertilizers, the protection of soil health in agroecosystems, and the integration of biogas residues into soil management, represents an essential pathway for circular and sustainable agriculture. Waste optimization and valorization should be prioritized as core strategies to preserve the sustainability of natural resources and ensure their intergenerational availability. Preserving the adaptive capacity of species to withstand climate variability, diseases, and pests, as well as safeguarding genetic and biological diversity, is critical. Maintaining traditional and locally adapted varieties ensures ecosystem stability and resilience under changing environmental conditions. Implementing food system resilience strategies—such as enhancing sustainable local production and consumption and promoting peri-urban and urban agriculture—can significantly mitigate the impacts of global climate change and strengthen regional food self-sufficiency.

Techniques such as mulching, groundcover management, and manual weed control should be further developed and adapted to preserve soil moisture, suppress weeds, and enhance soil carbon retention. The use of green manure, through the cultivation of cover crops or the collection of nutrient-rich plant biomass from marginal or forested areas, contributes to soil fertility restoration and organic matter enhancement.

Developing regionalized agricultural production systems that are environmentally sound and less dependent on external inputs is essential. This includes reversing the trend toward large-scale industrialization and supporting small-scale and community-based farming systems (Giller et al., 2021). The emergence of smaller urban centers in rural landscapes represents a promising trend that fosters local market-oriented production, short supply chains, and value-added opportunities in processing and agrifood entrepreneurship. Approaches such as organic farming, agroecology, circular and regenerative agriculture, urban farming, and nature-inclusive systems should be actively promoted to diversify production landscapes, strengthen smallholder participation, and enhanced ecological resilience. In parallel with biodiversity conservation and sustainable natural resource management, it is crucial to establish policies and regulatory frameworks that safeguard the rights, livelihoods, and well-being of local and indigenous communities. Enhancing food use efficiency through the reduction of food waste and postharvest losses can substantially alleviate food access challenges and strengthen the equity and sustainability of food systems (Sperling et al., 2022).

Food production systems that rely on fewer external inputs, use locally certified supply chains to serve local markets, and promote the diversification of crops toward more nutritious plant species should be strongly encouraged. Community gardens and urban food systems should be established on unused private and public lands, and a designated share of food purchased by public institutions should be sourced from local farms and food producers. Green cities and UA should be actively supported as key sources of local and sustainable food production. Municipal governments should prioritize making cities and their surroundings safe for food production, expanding healthy food enterprises, strengthening food safety networks, and ensuring access to healthy food and beverages. Community-controlled urban farms should be established within designated urban agricultural zones. In hot and arid regions near population centers, industrial-scale greenhouse cultivation techniques should be developed to produce high-value fresh vegetables with low land requirements and high nutrient and water-use efficiency, thereby reducing transportation costs and postharvest losses (Goddek et al., 2023).

Agricultural microbiotechnology and its related industrial applications should be supported and promoted across all sectors. Sustainable microbial technologies, utilizing beneficial microorganisms, should be integrated into agricultural practices, production systems, and bioenergy generation. Emerging synthetic biology strategies in agriculture can reduce fertilizer dependency, enhance plant nutritional value, yield, and carbon-use efficiency, and enable the development of next-generation bioproducts. Future agricultural innovation requires the design of stress-responsive genetic circuits through synthetic biology techniques (Lohani et al., 2022) and the development of dedicated energy crops to reduce competition between food and bioenergy production and to expand plant-based cell factory applications. However, achieving these goals requires extensive research on plant metabolic pathways, practical implementation planning, and the advancement of tissue culture methodologies (Liu et al., 2023). Future research should prioritize the development and regional optimization of sustainable agricultural practices, the integration of advanced PA technologies—including the IoT and AI— and the formulation of policies that support sustainable farming across all governance levels. Through the development of sustainable farming techniques and innovative management strategies, resource use efficiency can be optimized, crop productivity increased, and soil health enhanced. Future research should also emphasize health-promoting and cost-effective preventive strategies that strengthen human–nature interdependence and enhance overall well-being and quality of life.

## 12. Conclusion

The agricultural sector continues to expand with the adoption of emerging technologies such as precision agriculture (PA), smart farming, machine learning (ML), the internet of things (IoT), and advanced supply chain management; however, ensuring affordability and accessibility remains crucial to preventing smallholder farmers from being marginalized in this technological transformation. As smallholder farms will continue to produce a significant share of food in rural areas and ensure food security for a large proportion of the global population, it is critical to provide them with investment and infrastructure support while reducing their dependency on large-scale agriculture, despite the challenges involved. As a living and dynamic ecosystem, soil must be protected to maintain its structure, biological integrity, diversity, and capacity to sustain terrestrial productivity and ecosystem services. Resource-use efficiency should be enhanced, sustainable agricultural practices should be promoted, and environmental considerations should be fully integrated into resource management frameworks. In a world facing intensifying climate variability and accelerating resource depletion, environmental health and food and nutrition security will inevitably come under growing pressure; therefore, a rapid transition toward sustainable and resilient food systems is essential to mitigate risks to agricultural production and global food security.

## Figures and Tables

**Table 1:** Promising solutions, potential pathways, and innovations for transitioning toward sustainable agriculture and mitigating long-term challenges.

Promising solutions, potential pathways, and innovations	References
Agroecological approaches, sustainable intensification, precision farming technologies, integrated pest management, conservation agriculture, soil and sustainable water management practices	(Chowdhuri and Pal, 2025)
Agroecological practices, minimizing chemical inputs, promoting ecological balance; agroforestry, cover cropping, water harvesting	(Singh et al., 2024)
Crop rotation	(Yu et al., 2022)
Cropping system diversification and intensification	(Yadav et al., 2024)
Cover cropping, double and multicropping, livestock integration, nutrient cycling, and composting	(Croitoru et al., 2024)
Eco-efficiency and eco-effectiveness approach	(Taoumi and Lahrech, 2023)
Integrated digital technologies	(Gebresenbet et al., 2023)
Implementing sustainability-oriented innovation	(Little et al., 2023)
Improving input-use efficiency through methods such as precision farming, substituting inputs, and redesigning systems	(Therond et al., 2017; Soulé et al., 2023)
Regeneration of degraded areas	(Sperling et al., 2022)
Resource efficiency, systems diversification, sustainable bioeconomy	(Meybeck et al., 2024)
Adopting clean technology, renewable energy sources, and resource-efficient processes	(Luo et al., 2024)
Combining indigenous knowledge with advances in food science and technology for the sustainability of future food systems	(Knorr and Augustin, 2025)
Designing a sustainable cropping pattern	(Taheri et al., 2025)
Industrial symbiosis	(Haller et al., 2022)
Reduce the ecological footprint by focusing on mitigating climate change, adapting to its effects, and reversing biodiversity loss to achieve a positive environmental impact Sustainably improve agricultural productivity and increase crop production by achieving multiple harvests per year Protect and restore natural ecosystems, ensuring a sustainable natural resource base, limiting land occupation, reducing water consumption, and enhancing the efficiency of agricultural processes Reduce pollution, in particular pollution from waste generation and the emission of atmospheric pollutants, including greenhouse gases that contribute to global warming Reduce energy consumption and dependence, especially on fossil fuels, and transition to clean, renewable energies Minimize food loss and waste; shorten distribution chains and times Decrease the use of pesticides and excessive fertilization Promote the circular economy, organic farming, and diversity Minimize plant diseases, the proliferation of pests Promote healthier diets, encourage more plant-based protein intake Enhance the efficiency, inclusivity, and resilience of food systems Promote research, innovation, technology, and investment in food systems Ensure food security, nutrition, and public health Decrease the cost and improve accessibility of food products and healthy diets	(WRI, 2019; EC, 2020; EC, 2022; Calicioglu et al., 2019; Sousa et al., 2024)

**Table 2:** Resilient, resource-efficient, and sustainable alternative farming systems, methods, and practices.

Improving agricultural efficiency	References
Agroecology	(Garibaldi et al., 2017)
Agroforestry	(Cyamweshi et al., 2023; Fatima et al., 2024)
Agribased circular bioeconomy	(De Corato et al., 2024)
Agroforestry and biofertilizer, agroecological, and agroforestry strategies	(Silva-Galicia et al., 2023)
Agroecological and agroforestry strategies	(Silva-Galicia et al., 2023)
Agroecological agriculture	(Moldavan et al., 2023)
Agroecological intensification	(Thakur et al., 2022)
Agroecological management and land conversion	(Le et al., 2023)
Agroecological symbiosis	(Kujala and Koppelmäki, 2024)
Biointensive sustainable mini-farming	(Jeavons, 2001)
Carbon farming	(Sharma et al., 2021; Paul et al., 2023)
Climate-smart agriculture	(Waaswa et al., 2022; Feng et al., 2023)
Climate-smart regenerative agriculture	(Keshavarz and Sharafi, 2023)
Climate-resilient agriculture	(Zong et al., 2022)
Conservation agriculture, reduced tillage, and crop diversification	(Hoque et al., 2023)
Community supported agriculture	(Tay et al., 2024)
Digital agriculture	(MacPherson et al., 2022)
Diversified farming	(Jones et al., 2021)
Ecological intensification	Kovács-Hostyánszki et al., 2017)
Family farming	(Chao, 2024)
Local food systems	(Kamiyama et al., 2023)
Localized agrifood systems	(Burnett, 2023)
Long-term crop rotation	(Sehgal et al., 2023)
Hydroponic systems	(Sousa et al., 2024)
Industrial-scale greenhouse horticulture	(Goddek et al., 2023)
Integrated farming system models	(Kumar et al., 2018; Fatima et al., 2023)
Integrated crop–livestock–forestry systems	(Carvalho et al., 2024)
Integrative permaculture and regenerative agriculture	(McLennon et al., 2021)
Integrating perennial groundcovers Perennial groundcover systems	(Schlautman et al., 2021)
Innovative and resilient ecological farming systems	(Knickel et al., 2018)
Indoor smart gardens	(Mihailović et al., 2023)
Mixed fruit tree–vegetable systems	(De Lapparent et al., 2023)
Mulch cropping and cover cropping Changing cropping patterns and crop rotations Adaptation to agroclimatic regions Efficient water and irrigation management Integrated nutrient, pest, and weed management	(Mehraj et al., 2024)
Intercropping	(Yu et al., 2025)
Integrated nutrient management	(Sheng et al., 2023)
Mixed crop and livestock systems	(Garrett et al., 2020)
Nature-based solutions	(Albert et al., 2021)
Nitrogen fertilizer management	(Fabbri et al., 2023)
Natural pest management or integrated pest management (IPM)	(Green et al., 2020; Collier, 2023)
Organic farming	(Rempelos et al., 2021; Çakmakçı and Çakmakçı, 2023a)
Perennial groundcover systems	(Schlautman et al., 2021)
Process intensification technologies	(Chiang, 2024)
Tree-crop intercropping systems	(Yang et al., 2023)
Green manuring and residue retention	(Ansari et al., 2022)
Precision agriculture	(Karunathilake et al., 2023)
Site-specific management	(Shaheb et al., 2022)
Strip intercropping techniques	(Wu et al., 2023)
Sustainable intensification and agroecology	(Boix-Fayos and de Vente, 2023)
Sustainable intensification of cropping systems	(Yadav et al., 2024)
Controlled environment farming	(Ragaveena et al., 2021)
Tropical tree-based food production systems	(Jansen et al., 2020)
Urban agriculture	(Qu et al., 2024)
Zero tillage/no-till farming	(Wortmann and Dang, 2020)

## Footnotes

1 FAO (2022 a). Land Cover. FAOSTAT. FAO, Rome [online]. Website: fao.org/faostat/en/#data/LC [accessed 02 10 2025]

2 IPCC (2020). Intergovernmental Panel on Climate Change, 2020. 2019: Land–Climate Interactions. (2020). Climate Change and Land: an IPCC Special Report on Climate Change, Desertification, Land Degradation, Sustainable Land Management. Food Security, and Greenhouse Gas Fluxes in Terrestrial Ecosystems [online]. Website: ipcc.ch/report/ar6/wg1/#FullReport [accessed 01 10 2025].

3 UNIDO (2022). Circular Economy and Agribusiness Development. UNIDO. Vienna, Austria [online]. Website: unido.org/sites/default/files/files/2022-03/Circular economy and agribusiness development.pdf [accessed 01 10 2025].

4 FAO (2018). The State of Food Security and Nutrition in the World. Building Climate Resilience for Food Security and Nutrition. Rome [online]. Website: fao.org/3/i9553en/i9553en.pdf [accessed 02 10 2025]
